# Correction: Designing transformer oil immersion cooling servers for machine learning and first principle calculations

**DOI:** 10.1371/journal.pone.0275189

**Published:** 2022-09-20

**Authors:** PLOS ONE Staff

## Notice of Republication

An incorrect version of Fig 1 and Fig 2 was introduced during typesetting and published in error. This article was republished on August 1, 2022. The publisher apoligizes for the error. Please download this article again to view the correct version.
