# Supplementary material for: Designing transformer oil immersion cooling servers for machine learning and first principle calculations
Source: PLoS One. 2022 May 17;17(5):e0266880. doi: 10.1371/journal.pone.0266880 (PMC9113583; doi:10.1371/journal.pone.0266880)
Supplement: Attachment — Submitted filename: Comment.pdf [file pone.0266880.s001.pdf]

Computational servers are important in almost all fields of science and technology. These servers generate large amount of heat. Efficient cooling is required to keep the servers functional for a longer period of time. The performance of heated servers usually goes down. In this paper, authors have put their efforts in solving the issue of heated servers and have tried to use transformer oil as a heat sink. Authors have reported that speed of first principle calculations and machine learning applications are better in servers which are immersed in transformer oils. This is really a nice piece of work however authors are requested to clarify few doubts:

- Authors report that the server is functional in the oil. Did they use any type of coating on the server to protect the machine?
- Authors see an improvement in server's performance after keeping it inside the transformer oil. Authors should explain the reason behind this finding.
- Authors get better results in multi-core machines. The reason behind this observation is missing.
- It is also interesting to know if the same oil can be recycled again and for how many time.

The authors studied the effect of water molecules on the chlorination of alcohols using well known S_N2 mechanism. Few simple alcohols were taken for density functional theory-based investigations. It is shown in the study that few water molecules must be included near to the reaction site for solvating the nucleophile and the leaving group (Cl^-). 1-4 water molecules were considered in the computational model and static DFT was performed.

Suggestions:

1. The computational model used by the authors is too simple. Authors have ignored the water dynamics near to the reaction site. In fact, authors have shown in their simple model that it is better to consider a greater number of water molecules than taking no or one water molecule. Both, barriers and product stabilities are greatly affected by number of water units. Therefore, in order to understand precisely the solvation effect in the said reaction authors should use Hybrid QM/MM molecular dynamics simulations. They can build a box full of water and then study the chemical reaction. For help, Rocha and coworkers are recommended to go through the paper provided below.

Hybrid QM/MM molecular dynamics simulations for an ionic S_N^2 reaction in the supercritical water: OH^- + CH_3Cl --> CH_3OH + Cl^-. DOI: 10.1002/jcc.10134.

This is a two-decade old paper but has studied a very similar reaction. Authors can also look for citation of this paper.

2. Authors want to understand the stability of the nucleophile and the leaving group due to the microsolvation of water molecules. Thus, Grimme's dispersion correction must be added to

account mid-range and long-range interactions. It seems that dispersion is missing in the present work.

3. Authors are recommended to correlate their computational results with experimental data available in the literature. The studied reaction is a well-known reaction so finding such data would not be a challenge.

4. Authors have provided clear and clean data. Paper is easy to understand. The presentation is nice however authors should put more efforts in better quality figures. It is also recommended to proof read the manuscript as there are typos.

Typo> Page 3, line 54: Replace “in order to characterized” to “in order to characterize”

I believe that authors should include suggestions provided above in the revised manuscript.
